# Reduced progression of bone erosion in cytomegalovirus seropositive rheumatoid arthritis patients

**DOI:** 10.1186/s13075-020-2098-1

**Published:** 2020-01-20

**Authors:** B. Rauwel, Y. Degboé, D. Nigon, J.-F. Boyer, F. Abravanel, J. Izopet, B. Combe, A. Ruyssen-Witrand, A. Constantin, A. Cantagrel, J.-L. Davignon

**Affiliations:** 1 Centre de Physiopathologie Toulouse Purpan, U.1043 INSERM, CNRS, Bât A, CHU Purpan, BP 3028, 31024 Toulouse cedex 3, France; 2 Centre de Rhumatologie, CHU de Toulouse, Toulouse, France; 3 Faculté de Médecine, Université Paul Sabatier Toulouse III, Toulouse, France; 4 UMR1027, INSERM – Université Paul Sabatier Toulouse III, Toulouse, France; 5 CHU de Toulouse, Hôpital Purpan, Laboratoire de Virologie, Toulouse, France; 6 Département de Rhumatologie, CHU Montpellier, Université de Montpellier, Montpellier, France

**Keywords:** Human cytomegalovirus, Rheumatoid arthritis, Bone erosion, Inflammation, ESPOIR cohort

## Abstract

**Background:**

Human cytomegalovirus (HCMV) seropositivity has been associated with higher inflammation during rheumatoid arthritis (RA). However, no data are available on the impact of HCMV seropositivity on bone erosion progression during RA.

**Methods:**

We selected 487 individuals of ESPOIR cohort who fulfilled the 2010 ACR/EULAR criteria for RA. HCMV serology for these patients was determined using Architect CMV IgG assay. Baseline and 1-year central X-ray reading using modified Total Sharp Score (mTSS), Erosion Sharp Score, and joint space narrowing Sharp score were used to quantify structural damage progression. We performed univariate and multivariate analyses to investigate the association between HCMV status and bone erosion progression.

**Results:**

We analyzed 273 HCMV seropositive (HCMV+) and 214 HCMV seronegative (HCMV−) RA patients. At inclusion, HCMV+ patients were less frequently ACPA+ (49.8% versus 58.9%, *p* < 0.0465) and had a higher DAS28-ESR (5.55 ± 1.24 versus 5.20 ± 1.14, *p* < 0.0013) in comparison with HCMV−. At 1 year, bone erosion progression (delta erosion Sharp score > 1 point) was lower in HCMV+ patients (16.1% versus 25.2%, *p* = 0.0128) in comparison with HCMV−. HCMV+ status remained independently associated with lower bone erosion progression in multivariate analysis.

**Conclusions:**

Our findings suggest that, independently of other confounding factors, HCMV seropositivity is associated with a lower progression of bone erosion during RA.

## Background

Rheumatoid arthritis (RA) is a complex disease resulting of an interaction between genetic factors involved in immunity, environmental events, and epigenetic modifications. Among environmental factors, smoking and infectious agents such as *Porphyromonas gingivalis* are well described and associated with anti-CCP production [1]. However, other infectious agents such as viruses also could have an impact on RA pathophysiology.

In 2012, Pierer et al. analyzed the relationship between human cytomegalovirus (HCMV) infection and RA [2], based on the Steinbrocker radiographic score at a single time point after several years of disease. Their study indicated that a positive serology for HCMV infection is associated with a more severe clinical course of RA.

Recently, our laboratory discovered, in vitro, that HCMV infection is able to inhibit osteoclastogenesis through inhibition of CSF-1R expression [3], which prompted us to consider that HCMV could have an impact on joint destruction evolution during early RA, and more particularly on bone erosion. Using a different approach from a large national prospective cohort, we asked instead whether HCMV could play a role in the evolution of bone erosion in RA. To this end, we chose to evaluate the radiographic van der Heijde-modified Sharp score over 1 year of evolution in ESPOIR cohort of early RA patients.

## Methods

### ESPOIR cohort

“ESPOIR” is a multicenter, longitudinal, prospective cohort of 813 French patients with early arthritis. The characteristics of the cohort have been described previously [4]. Briefly, 813 patients with early arthritis recruited in 14 centers in France with arthritis duration < 6 months and no prior treatment with disease-modifying antirheumatic drugs were included in the cohort between 2002 and 2005. Patients underwent clinical, biological, and radiological assessments at baseline and at each subsequent visit. Local institutional review boards approved the study, and written informed consent was obtained from all participants in the study.

Dosages of CRP, IgA, and IgM rheumatoid factor and ACPA were performed. Baseline and 1-year central X-ray reading (feet and hands) using modified total Sharp score (mTSS), erosion Sharp score (ESS), and joint space narrowing Sharp score (NSS) were performed by a single experienced rheumatologist (C. Lukas). Intraclass correlation coefficient was calculated from a random sample of 30 radiographs scored twice and was about 0.99 [5]. The smallest detectable change (SDC) was calculated at 1.0 mTSS unit and was derived from distribution-based methods and duplicate reading experience, as explained in the listed reference [5]. Formally, this SDC is thus “the smallest change that can be detected by the instrument beyond measurement error,” and patients in whom the change was scored beyond this cutoff value should thus be regarded “real progressors,” even though the clinical relevance of such a small value can be discussed at the individual level.

### Patients and HCMV serology

Among the 813 patients included in the ESPOIR cohort, 487 fulfilled the 2010 American College of Rheumatology/European League Against Rheumatism criteria for RA at baseline [6] with a complete dataset of van der Heijde-modified Sharp score radiographic evaluation at baseline and 1 year. HCMV serology for these patients was determined using Architect HCMV IgG assay (Abbott, Chicago, IL, USA).

### Statistical analysis

The Shapiro-Wilk test was performed to assess the normality of continuous data, presented as mean (SD) if normal or median (inter-quartile range [IQR]) else. Qualitative variables are presented as number (percentage).

Comparisons of normally distributed data according to HCMV status were performed with Student test, non-Gaussian variables with Mann-Whitney, and dichotomous variables with *χ*^2^ test (or Fisher’s exact test if the contingency table contains an observed number of occurrences inferior to 5). Odds ratio (OR) was calculated and presented with 95% confidence interval (95% CI) to show the association strength between HCMV status and radiological evolution.

Variable to explain was the presence of more than one new erosion at 1 year in relation to baseline. All the candidate explanatory variables were selected in univariate analysis with *α* = 20%. A downward logistic regression with *α* = 5% was then carried out, to identify covariates independently associated with the variable to explain.

All tests performed for comparison were two-tailed, with *p* < 0.05 considered statistically significant.

Data were analyzed with Stata IC 12.1 (StataCorp, College Station, Texas).

## Results

Sera from 487 individuals who fulfilled the 2010 ACR/EULAR criteria for RA were analyzed for anti-CMV IgG antibodies. A total of 214 patients (44%) were seronegative and 273 (56%) seropositive for HCMV. At inclusion, HCMV+ patients were found to be significantly older than HCMV− patients (median 52.9 years for HCMV+, 47.8 years for HCMV−, *p* = 0.0001). In addition to being older, HCMV+ population patients presented a lower proportion of ACPA+ (49.8% versus 58.9%, *p* < 0.0465) and a higher DAS28-ESR (5.55 ± 1.24 versus 5.20 ± 1.14, *p* < 0.0013). No additional significant difference was observed between HCMV+ and HCMV− patients at inclusion (Table 1).

**Table 1:** Characteristics of ESPOIR RA patients at inclusion

	ESPOIR cohort			
	All RA patients (*n* = 487)	HCMV seropositive RA patients (*n* = 273) (56%)	HCMV seronegative RA patients (*n* = 214) (44%)	*p* (HCMV+ versus HCMV−)
Baseline characteristics (inclusion)				
Age, years, median (IQR)***	50.3 (40.0–57.1)	52.9 (43.1–58.5)***	47.8 (37.4–54.3)	0.0001
Gender, female, *n* (%)	378 (77.6)	219 (80.2)	159 (74.3)	0.1197
Symptom duration, year, median (IQR)	0.42 (0.26–0.64)	0.41 (0.25–0.62)	0.42 (0.27–0.65)	0.5108
ACPA+, *n* (%)*	262 (53.8)	136 (49.8)*	126 (58.9)	0.0465
RF+, *n* (%)	296 (60.8)	163 (59.7)	133 (62.1)	0.5837
Disease Activity Score 28 (DAS28-ESR), mean (IQR)*	5.40 (± 1.21)	5.55 (± 1.24)*	5.20 (± 1.14)	0.0013
Erythrocyte sedimentation rate (ESR), median (IQR)	24 (12–39)	24 (14–46)	22.5 (10.5–35.5)	0.0566
C-reactive protein (CRP), median (IQR)	10 (3–24)	9 (3–24)	11 (3–24)	0.5510
Total Sharp score (TSS), median (IQR)	4 (1–8)	4 (1–8)	3 (1–8)	0.6745
Erosion Sharp score (ESS), median (IQR)	1 (0–4)	1 (0–4)	1 (0–4)	0.5420
Joint space narrowing Sharp score (NSS), median (IQR)	1 (0–4)	1 (0–4)	1 (0–4)	0.9121

* *p* < 0.05; *** *p* < 0.001

After 1 year, HCMV+ and HCMV− did not display significant differences in therapy administration. DMARDs and glucocorticoids were similarly administrated in both populations. Although mTSS was not significantly different between HCMV+ and HCMV− patients after 1 year, progression of this score was lower in HCMV+ patients: 17.2% of HCMV+ patients had a delta total Sharp score > 1 in comparison with 26.3% of HCMV− (*p* = 0.0151) (Table 2).

**Table 2:** Treatments and disease characteristics of ESPOIR RA patients 1-year post-inclusion

	All RA patients (*n* = 487)	HCMV seropositive RA patients (*n* = 273) (56%)	HCMV seronegative RA patients (*n* = 214) (44%)	*p* (HCMV+ versus HCMV−)
Patients with DMARD, *n* (%)	417/458 (91.05)	237/262 (90.5)	180/196 (91.8)	0.6090
Use of csDMARD, *n* (%)	376/458 (82.10)	210/262 (80.15)	166/196 (84.70)	0.2100
Use of bDMARD, *n* (%)	41/458 (8.95)	27/262 (10.31)	14/196 (7.14)	0.2410
Delay between inclusion and first DMARD (months), median (IQR)	0.38 (0.03–1.20)	0.39 (0.03–1.22)	0.36 (0.07–1.38)	0.5225
Patients with glucocorticoids at baseline, *n* (%)	0 (0)	0 (0)	0 (0)	–
Cumulated dose of glucocorticoids between inclusion and 1 year in milligram, median (IQR)	210 (0–2120)	646 (0–2217)	100 (0–1935)	0.1393
Total Sharp score (mTSS), median (IQR)	4 (1–10)	4 (1–9)	4 (1–10)	0.8913
Delta total Sharp score (0–1 year), mean (SD)*	1.70 (4.49)	1.29 (3.38)	2.22 (5.57)	0.0238
Delta total Sharp score (0–1 year), median (IQR)	0 (0–1) p95 = 11	0 (0–1) p95 = 9	0 (0–2) p95 = 13	0.0349
		OR [95% CI] = 0.623 [0.464–1.005]		0.0540
Delta Sharp total score (0–1 year) > 1, *n* (%)*	103 (21.2)	47 (17.2)*	56 (26.3)	0.0151
		OR [95% CI] = 0.583 [0.367–0.925]		0.0151
Joint space narrowing Sharp score (NSS), median (IQR)	1 (0–5)	1 (0–5)	1 (0–4)	0.9121
Delta joint space narrowing Sharp score (0–1 year), mean (SD)	0.26 (1.16)	0.24 (1.17)	0.29 (1.14)	0.6992
Delta joint space narrowing Sharp score (0–1 year), median (IQR)	0 (0–0) p95 = 2	0 (0–0) p95 = 1	0 (0–0) p95 = 2	0.4129
		OR [95% CI] = 0.763 [0.378–1.541]		0.4129
Joint space narrowing Sharp score (0–1 year), Δ > 1, *n* (%)	26/486 (5.3)	13 (4.8)	13 (6.1)	0.5140
		OR [95% CI] = 0.769 [0.321–1.846]		0.5140
Erosion Sharp score (ESS), median (IQR)	2 (0–5)	2 (0–5)	2 (0–6)	0.9588
Delta erosion Sharp score (0–1 year), mean (SD)*	1.43 (3.82)	1.05 (2.63)	1.93 (4.90)	0.0112
Delta erosion Sharp score (0–1 year), median (IQR)*	0 (0–1) p95 = 9	0 (0–1) p95 = 8*	0 (0–2) p95 = 12	0.0172
		OR [95% CI] = 0.637 [0.432–0.939]		0.0230
Erosion Sharp score (0–1 year), Δ > 1, *n* (%)*	98 (20.1)	44 (16.1)*	54 (25.2)	0.0128
		OR [95% CI] = 0.569 [0.355–0.911]		0.0128

* *p* < 0.05

**Fig. 1:**
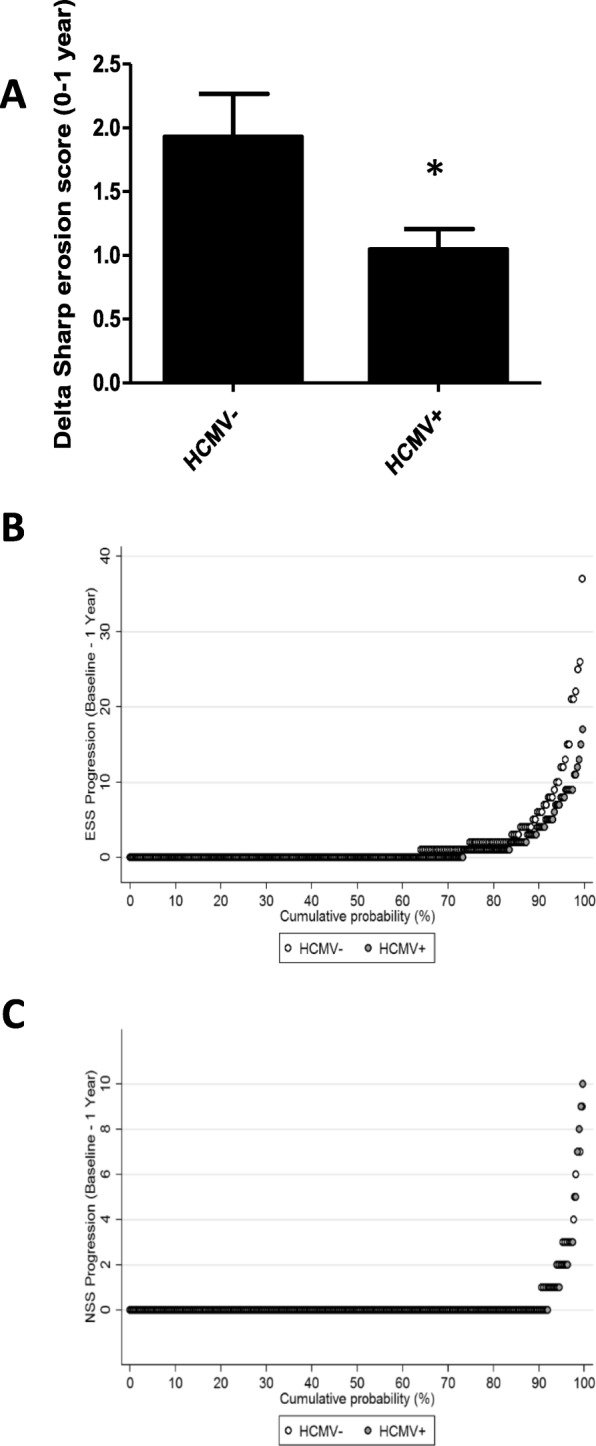
HCMV seropositivity is associated with a lower bone erosion progression during rheumatoid arthritis, study of ESPOIR cohort. **a** Delta erosion Sharp score evolution (0–1 year) is represented for 214 HCMV− and 273 HCMV+ RA patients from the ESPOIR cohort. (*n* = 487, *p < 0.05, error bars as SD). **b**, **c** Cumulative probability plots of the progression of ESS (erosion Sharp score) and NSS (joint space narrowing Sharp score) within 1 year according to HCMV status

When mTSS was split into joint space narrowing Sharp score (NSS) and erosion Sharp score (ESS), we observed that difference of delta mTSS was only related to ESS change. Indeed, no significant difference was observed on delta NSS between HCMV+ and HCMV− patients (4.8% of HCMV+ patients had a delta NSS > 1 in comparison with 6.1% of HCMV−, *p* = 0.5140) (Table 2). However, when looking specifically at ESS, we found that the proportion of patients with delta ESS > 1 was significantly lower in of HCMV+ patients (16.1%) as compared to HCMV− patients (25.2%) (*p* = 0.0128) (Table 2). Among these patients, 10.3% of HCMV+ patients (*n* = 28) and 14% of HCMV− patients (*n* = 30) had a delta ESS > 3 (*n* = 58 patients; *p* = 0.2033). ESS progression was 2-fold lower in HCMV+ patients in comparison with HCMV− (Fig. 1a), showing an association between HCMV seropositivity and a lower bone erosion progression during RA.

Furthermore, cumulative probability plot showed that ESS was less pronounced in HCMV+ patients (Fig. 1b). NSS was not significantly different (Fig. 1c).

In order to identify covariates independently associated with delta Sharp erosion score > 1, we performed a multivariate analysis (including parameters associated in univariate analysis with *α* = 20%: age, gender, ACPA, RF, HCMV, DAS28-ESR, CRP, duration of disease course before inclusion, smoking consumption, glucocorticoids treatment, erosion Sharp score at baseline, bDMARD, and csDMARD). ACPA+ status was more frequent in HCMV− patients. We forced the multivariate model with the use of a bDMARD or a csDMARD at year 1. The results demonstrated that while ACPA were, as expected, associated with progression of erosion, HCMV seropositivity was nevertheless still associated with lower progression (Table 3, OR = 0.5255, CI = 0.2998 to 0.9213, *p* = 0.025).

**Table 3:** Multivariate analysis of the association between HCMV seropositivity and ESS progression during RA, study of ESPOIR cohort

Erosion Sharp score (baseline–1 year) score Δ > 1	Odds ratio	Standard error	*z*	*P* > lzl	[95% conf. interval]	
Erosion Sharp score at baseline	5.3180	1.9356	4.59	0.000	2.6057	10.854
ACPA+	5.1915	1.7635	4.85	0.000	2.6677	10.103
HCMV	0.5255	0.1505	− 2.25	0.025	0.2998	0.9213
Cumulated dose of glucocorticoids	0.9997	0.0001	− 2.29	0.022	0.9995	1
Use of csDMARDs	1.7987	1.0696	0.99	0.324	0.5608	5.7694
Use of bDMARDs	1.3298	0.9829	0.39	0.700	0.3123	5.6619

## Discussion

Although HCMV+ status was previously described to be associated with more severe joint disease [2], we show here, from a large cohort of early RA patients, that HCMV seropositivity is associated with a lower progression of bone erosion in the first year of the disease despite higher DAS.

This discrepancy could result from a longer history of RA disease in patients evaluated in Pierer et al. compared with RA patients in the first year of the disease used in our current work. Analysis of joint destruction was also different as Steinbrocker score analyzes qualitative bone destruction, contrary to the Sharp-van der Heijde score which gives a quantitative measurement of joint destruction and can discriminate joint space narrowing and bone erosion. Finally, joint damage was measured at a given time point in Pierer et al., whereas the ESPOIR cohort was designed for analysis of progression.

Overall, apparent contradiction between higher DAS and lower bone erosion in HCMV+ patients can be pointed out in our current study. This could result from dissociation of HCMV-induced inflammation [7] from its specific effect on bone erosion observed here over 1 year. In this respect, HCMV may indeed aggravate RA disease over time. The Steinbrocker score used in the paper by Pierer et al. that is not specific for bone erosion may reflect the inflammatory status as a whole at a given time point. Conversely, the 1-year Sharp erosion score as in our current study of the ESPOIR cohort may best evaluate the evolution of specific bone degradation at the early phase of the disease. Although the average change in radiographic damage that was observed in the ESPOIR cohort was limited, which might be due to an overall milder disease, early therapeutic interventions and close follow-up, or even more probably a combination of such effects, it must be accepted that the changes that were observed and scored, although of limited amplitude and in occurring a restricted population only, are definitely real. Our observations were based on a delta ESS > 1. Nevertheless, we performed the analysis with a cutoff of 3 and observed a non-significant smaller proportion of HCMV+ RA patients (10.3% versus 14%, *p* = 0.2033). This result may be related to a lack of statistical power due to the small number of patients (*n* = 58). Our study provides some insight in the population of patients with low ranges of the progression score. Our findings need to be confirmed in a population with higher ranges of progression score.

Since HCMV seropositivity relies on the presence of IgG antibodies in sera of RA patients, it is impossible to know when the primo-infection occurred in patients. HCMV, which establishes a lifelong persistence in the human host, is in a latent state in seropositive individuals, including RA patients. How latent HCMV infection, during which no viral protein is expressed, leads to decreased bone erosion is unknown. Since HCMV can reactivate from latency during monocytes differentiation into dendritic cells or macrophages, we can hypothesize that local reactivation in the synovium can lead to inhibition of OCs differentiation. It has been repeatedly shown that HCMV can reactivate in various organs and can be released in fluids [8]. Whether synovial membranes of HCMV+ RA patients are a site of viral reactivation is to be determined through the measurement of viral copy numbers ex vivo. Indeed, HCMV DNA was found infrequently in the synovial fluids of RA patients [9, 10]. Since inflammation has been associated with reactivation [11], it is possible that a proportion of RA patients reactivate their latent HCMV during disease flare. However, other mechanisms can be envisaged as well. Alternatively, cellular protein(s), induced during the primo-infection that would remain expressed throughout the course of the disease, may result in less severe bone erosion. Those hypotheses will have to be explored to explain how RA patients are protected from bone erosion. In this respect, diminished expression of CSF1-R in cells infected by HCMV has been reported by Frascaroli et al. [12]. This may explain why HCMV infection inhibits the differentiation of monocytes into OCs [3]. We have further identified an mRNA-binding protein that is induced by HCMV infection and directly inhibits the expression of CSF1-R. Its expression in RA patients and its contribution to protection from erosion is not known. Additional file 1: Figure S1 gives a tentative insight into how we envisage the impact of HCMV on inflammation and bone erosion. How this protein is relevant to inhibition of bone erosion specifically in RA patients is to be further investigated.

## Conclusions

Our report suggests that during RA, HCMV is associated with a lower bone erosion progression while contributing to inflammation.

## Supplementary information

**Additional file 1:** **Figure S1.** HCMV infection inhibits the expression of CSF-1R, thus providing a putative mechanism for the reduced progression of erosion in seropositive patients. (PPTX 196 kb)

## Data Availability

All data and material concerning ESPOIR cohort were available on the website http://www.lacohorteespoir.fr/

## Abbreviations

HCMV: Human cytomegalovirus
ACR: American College of Rheumatology
EULAR: European League Against Rheumatism
RA: Rheumatoid arthritis
mTSS: Modified total Sharp score
NSS: Joint space narrowing Sharp score
ESS: Erosion sharp score
ACPA: Anti-citrullinated protein antibody
CRP: C-reactive protein
IQR: Inter-quartile range
OR: Odd ratio
IC: Confidence interval
DMARD: Disease-modifying anti-rheumatic drugs
ESR: Erythrocyte sedimentation rate
RF: Rheumatoid factor
