# Supplementary material for: Reduced progression of bone erosion in cytomegalovirus seropositive rheumatoid arthritis patients
Source: Arthritis Res Ther. 2020 Jan 20;22:13. doi: 10.1186/s13075-020-2098-1 (PMC6971916; doi:10.1186/s13075-020-2098-1)
Supplement: Supplementary file 1 — Additional file 1: Figure S1. HCMV infection inhibits the expression of CSF-1R, thus providing a putative mechanism for the reduced progression of erosion in seropositive patients. (PPTX 196 kb) [file 13075_2020_2098_MOESM1_ESM.pptx]

## Slide 1
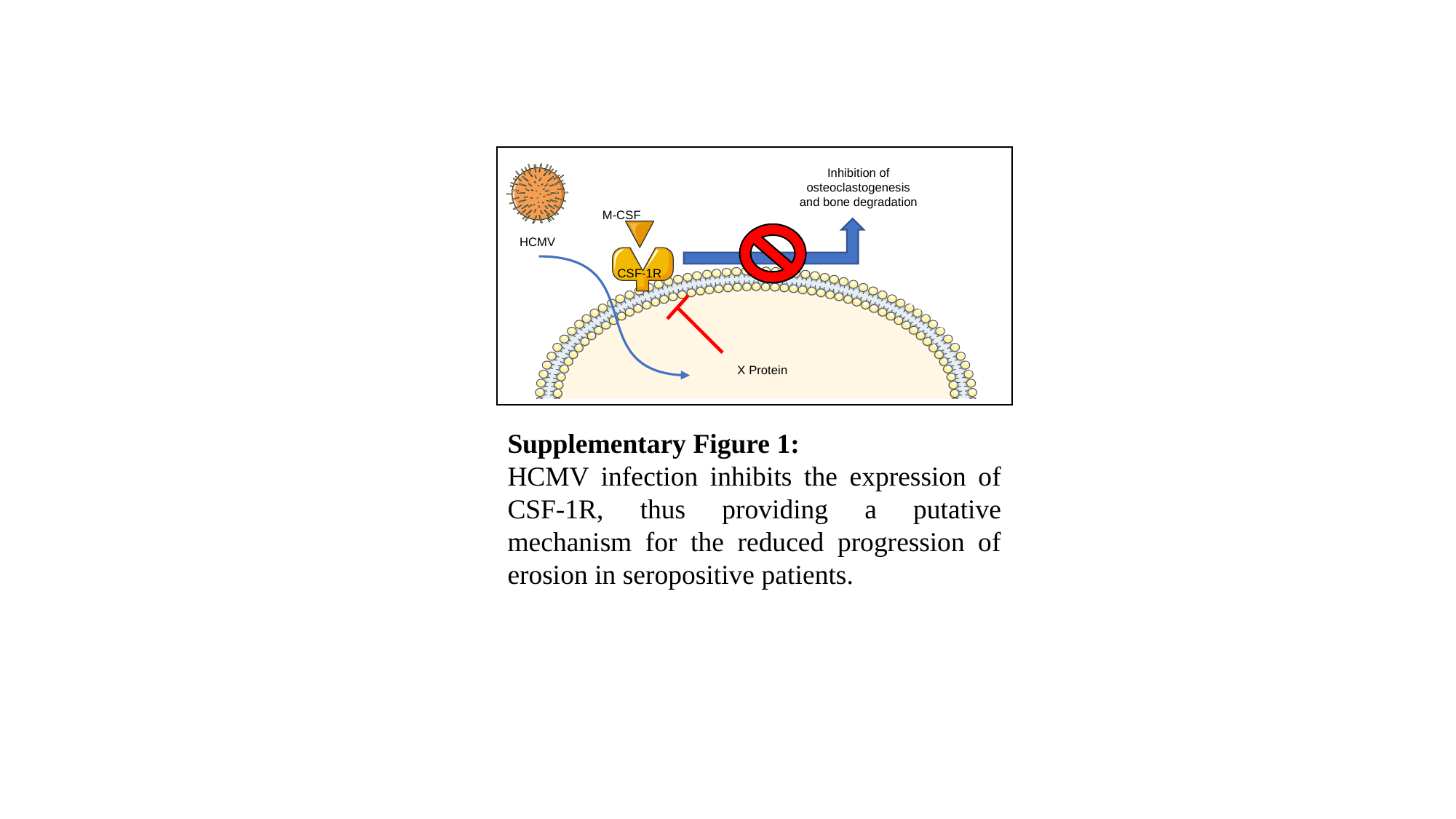

Inhibition of osteoclastogenesis
and bone degradation
M-CSF
HCMV
CSF-1R
X Protein
Supplementary Figure 1:
HCMV infection inhibits the expression of CSF-1R, thus providing a putative mechanism for the reduced progression of erosion in seropositive patients.
